# Effects on Some Therapeutical, Biochemical, and Immunological Parameters of Honey Bee (*Apis mellifera*) Exposed to Probiotic Treatments, in Field and Laboratory Conditions

**DOI:** 10.3390/insects11090638

**Published:** 2020-09-17

**Authors:** Ivana Tlak Gajger, Josipa Vlainić, Petra Šoštarić, Janez Prešern, Jernej Bubnič, Maja Ivana Smodiš Škerl

**Affiliations:** 1 Department for Biology and Pathology of Fish and Bees, Faculty of Veterinary Medicine, University of Zagreb, 10 000 Zagreb, Croatia; petrasos18@gmail.com; 2 Division of Molecular Medicine, Laboratory for Advanced Genomics, Institute Ruđer Bošković, 10 000 Zagreb, Croatia; Josipa.Vlainic@irb.hr; 3 Agricultural Institute of Slovenia, 1000 Ljubljana, Slovenia; janez.presern@kis.si (J.P.); jernej.bubnic@kis.si (J.B.); Maja.Smodis.Skerl@kis.si (M.I.S.Š.)

**Keywords:** honey bee colonies, probiotic treatments, field experiment, laboratory-controlled experiment, EM^®^ PROBIOTIC FOR BEES, honey bee physiology, nosemosis

## Abstract

**Simple Summary:**

Various negative factors contribute to a decline in insect pollinators. The aim of this study was to assess the impact of commercial probiotic EM^®^ PROBIOTIC FOR BEES on honey bees. The study was conducted in field and laboratory-controlled conditions. In the field, the sugar syrup with 10% of probiotic was administered by spraying or feeding the honey bee colonies in order to evaluate the colonies’ strength and *Nosema* spp. infection levels. In the laboratory, the adult workers have been fed with sugar syrup supplemented with 2.5, 5, and 10% of EM^®^ for bees for biochemical and immunological analyses of hemolymph, and with 5 and 10% for measuring the size of hypopharyngeal glands. It was found that following the EM^®^ for bees administration the *Nosema* spp. spore counts in colonies were significantly reduced, and colonies’ strength was increased. The results at the individual level showed positive physiological changes in treated groups of adult bees, but, at the same time, a higher mortality rate. Our findings indicate that the EM^®^ for bees is a promising food additive for nosemosis combating. Therefore, additional emphasis needs to be placed on studies investigating the nutritional requirements crucial to improve and sustain honey bee colonies health.

**Abstract:**

Several negative factors contribute to a decline in the number of insect pollinators. As a novel approach in therapy, we hypothesize that the EM^®^ for bees could potentially have an important therapeutic and immunomodulatory effect on honey bee colonies. The aim of our study was to evaluate its impact on honey bees at the individual and colony level. This is the first appliance of the commercial probiotic mix EM^®^ PROBIOTIC FOR BEES in honey bees as economically important social insects. The sugar syrup with 10% of probiotic was administered by spraying or feeding the honey bee colonies in the field conditions, in order to evaluate the infection levels with spores of *Nosema* spp. and colonies’ strength. Moreover, in laboratory-controlled conditions, in the hoarding cages, adult workers have been fed with sugar syrup supplemented with 2.5, 5, and 10% of EM^®^ for bees for biochemical and immunological analyses of hemolymph, and with 5 and 10% for measuring the size of hypopharyngeal glands. It was found that following the EM^®^ for bees administration the *Nosema* spp. spore counts in colonies were significantly reduced, and colonies’ strength was increased. The results at the individual level showed significant positive physiological changes in treated groups of adult bees, revealing at the same time a higher mortality rate when feeding sugar syrup supplemented with the probiotic.

## 1. Introduction

An alarming decline of insect pollinators’ population has been reported globally in the recent years [1,2,3]. The honey bees, among them, are probably the most important species from an ecological and economical aspect [4]. Pollination with honey bees has become a crucial component in agriculture technology [5,6]. Several environmental factors are suspected to have a negative impact on their colony strength and probability of their survival. Multiple causal factors are considered to contribute to colony losses: parasites and pathogens as the main candidates [3,7], exposure to pesticides [8,9,10,11], diet quantity, quality and diversity [12,13], as well as unfavorable weather and forage circumstances. Moreover, the health status of honey bee colonies is highly influenced by the beekeeper’s management practices [14,15]. All of these factors affect honey bee colonies individually or in various combinations [16], possible causing severe disturbance of honey bee microbiota composition. The honey bee hindgut contains a highly consistent bacterial community of six to nine species clusters which comprise the core of gut bacteria integrated in the host’s physiological ecosystem and could alter with the age of adult honey bees [17,18]. Adult honey bee dysbiosis (gastrointestinal microbial imbalance) is linked to a lower body weight, deficient development, and early workers mortality [19]. Furthermore, the altered microbiota is associated with host deficiencies. In such situation, the environmental stressors could change the gut bacterial balance and lead to visible manifestation of opportunistic diseases and other issues linked with honey bee colony declines [20]. Among them the increased number of *Nosema* spp. spores in the midgut, premature foragers senescence, and immune suppression linked with oxidative stress were reported [21].

Probiotic microorganisms compete with pathogenic microbes in the gastrointestinal tract of honey bees in adhesion to the intestinal epithelium [22]; protection against pathogens [19]; effects on immune response and metabolism [23,24,25,26,27,28], as well as impact to growth, development, and survival rates [19,29]. The presence of probiotics can result in a better availability and utilization of nutrients in honey bees. Published studies vary in their approaches concerning the effect of commercial probiotics and prebiotics on honey bee health. Some data have shown that probiotics increase honey bee mortality and pathogen loads, whereas others suggest that the administration of probiotics has an excellent effect on the strength of honey bee colonies, wax gland development, honey production, and protection against diseases [30,31,32,33,34,35,36,37]. The strains of probiotic bacteria that have been mostly used as a novel option for the management of honey bee microbiome were *Bifidobacterium* spp. and *Lactobacillus* spp. [32,38].

Nosemosis type C caused by the microsporidium *Nosema ceranae* adversely affects the honey bee health and can result in the complete colony collapse. Further studies on the potential treatment or beekeeping techniques are urgently required to combat the rapid spread of this dangerous disease. The role of this pathogen in global colony losses remains controversial [39,40], but a shortened lifespan due to the energetic stress of individual adult bees, consequent decreased colony strength, and even possible sudden collapses have been noticed [41,42]. *Nosema* spp. primary parasites and replicates within the epithelium of midgut and consequently impairs digestion and absorption of nutrients [43]. The result of the described parasitism is exacerbated nutritional stress where a causative agent relies on the host to furnish energy for reproduction and growth [44]. A recently published study has shown the correlation between nutrition-related gut bacterial dysbiosis in *A. mellifera* and the *N. ceranae* infection [19]. A negative influence of the mentioned factors on honey bee production and beekeeping management or profitability in general, have driven the development of sustainable alternative strategies for nosemosis type C therapy. The antibiotic fumagillin has been widely used in the treatment of nosemosis for a few decades [45]. However, as recently reported, fumagillin may exacerbate, rather than suppress, *N. ceranae* infection [46]. In addition, the use of antibiotics in the treatment of apian diseases is currently not approved by the European Union regulations. Today, there is not a single registered authorized veterinary medicine product (VMP) for nosemosis control and therefore the need for alternative therapies is bigger than ever.

EM^®^, commercially available as EM^®^ PROBIOTIC FOR BEES (hereinafter, EM^®^ for bees), is a proprietary probiotic formulation owned and managed by the EM Research Organization in Okinawa, Japan. It contains multiple species of lactic acid bacteria, yeast, and photosynthetic bacteria. As a novel approach in therapy, we hypothesize that EM^®^ for bees could have an important therapeutic and immunomodulatory effect at the individual and colony level. The aim of the current study was to evaluate the impact of multiple supplemental feedings of naturally diseased honey bee colonies in apiary conditions, on *Nosema* spp. infection levels. The obtained results were related with the strength of experimental colonies. At the same time, in laboratory-controlled conditions, several biochemical and immunological parameters were measured in hemolymph and hypopharyngeal glands (HPGs) of newly emerged workers in cages, after multiple supplemental feedings. This is the first appliance of commercial probiotic mix EM^®^ for bees in honey bees as economically important social insects.

## 2. Materials and Methods

### 2.1. Field Test

#### 2.1.1. Locations of Experimental Apiaries and Field Trail Design

The field part of the experiment was conducted during 40 consecutive days (beginning at 1 July 2019) at the apiary situated in the continental part of Croatia (45°56′54.71″ N, 16°37′46.06″ E), and according to the National classification space units for statistical needs NUTS 2–HR04 and NUTS 3–HR045, after the main harvesting season. In order to perform the field test, approximately 12 homogeneous honey bee colonies (*Apis mellifera carnica*, Pollmann, 1879) naturally infected with *Nosema* spp. and accommodated in standard Langstroth Root (LR) hives acquired from the same beekeeper, were selected and divided into experimental (2 × 4) and control (2 × 2) groups. At the beginning of the study none of the colonies showed clinical signs of brood diseases and the last treatment against the mite *Varroa destructor* was carried out at 20 June 2019 (CheckMite^®^, a.m. coumaphos) to avoid the negative effects of mite parasitation on colony health. No insecticides were used in the surrounding area during the experiment.

#### 2.1.2. Supplemental Feed Treatments and Adult Honey Bees Sampling

Honey bee colonies were additionally fed with a 0.25 L sugar syrup (1:1 water-sugar; Virosecer, Virovitica, Croatia) supplemented with an experimental concentration of 10% of EM^®^ for bees, every second day. The supplemented sugar syrup, as well as the pure sugar syrup, were administered to honey bee colonies as follows: experimental colonies (1–4) by spraying directly on frames covered with adult honey bees (a), and in feeders situated under the roof of the hives (5–8) (b), consecutively during 21 days. The pertaining control groups (control a, b) received only 0.25 L of sugar syrup prepared and provided in the same described way. The dose was adapted according to the manufacturer’s instructions. During the clinical inspection of honey bee colonies, approximately 60 forager bees *per* colony were collected from the hive entrance for a microscope examination on the presence of *Nosema* spp. spores. Adult bee samples were collected into clean plastic receptacles by catching the bees in front of hives entrances directly or using long tweezers. Each sample consisted of approximately 60 specimens (foragers) taken on the 10th (control II, EM IIa/EM IIb), 20th (control III, EM IIIa/EM IIIb), 30th (control IV, EM IVa/EM IVb), and 40th (control V, EM Va/EM Vb) day after the initial sampling (conducted prior to the first feeding session; control I, EM Ia/EM Ib).

#### 2.1.3. Examinations and Estimating the Strength of Honey Bee Colonies

Clinical signs of diseases, the presence of queen and mortality of the honey bees were checked on every inspection of the honey bee colonies at the experimental apiary. The Liebefeld method for visual determination of the number of adult bees as well as the brood amount was performed to estimate the strength of honey bee colonies [47], for experimental *vs.* control groups. The assessment of honey bee colony strength was conducted on the 1st (I) and 40th (II) day of the experiment, during morning hours, (9:00 to 10:00 a.m.), before the first massive forage flights of bees. For an easier assessment of comb areas covered with bees or brood, the frame for the LR hive divided with a plastic grid to 1 × 1 dm quadrants was used.

#### 2.1.4. Determination of *Nosema* spp. Infection Levels

Honey bees were counted in each sample; their abdomens were separated, thoroughly crushed, and homogenized in a plastic container loaded with 1 mL of pure water *per* one bee specimen. *Nosema* spp. spores were counted in each sample using a Malassez hemocytometer, and the infection levels were calculated according to the Office International des Epizooties guidelines (OIE) [48]. Each counting procedure was repeated three times. The counting equipment was carefully washed after each sample counting in order to avoid contamination with spores from the previous sample.

### 2.2. Trials in Laboratory-Controlled Conditions

#### 2.2.1. Trial Design in Incubators and Sampling of Adult Bees

For the trial in the laboratory conditions we collected five frames with a sealed brood from five honey bee colonies (*A. m. carnica*) kept at the Agricultural institute of Slovenia in Ljubljana, Slovenia. The colonies were free of *Nosema* spp. spores and showed no clinical signs of any disease. The frames were put in an incubator at 34 °C and left overnight. The plastic cake-like CD-storage boxes (~8 cm (H) × ~12 cm (dia.)) were prepared by drilling ~80 circular ventilation holes (each ~2 mm wide), in the top cover. Moreover, two additional holes (12 mm diameter) were added as place-holders for plastic feeding tubes. The next day we collected newly emerged honey bees and put them in the boxes, (~50 adult bees in each box). There were three treated groups of bees feed with the sugar syrup supplemented with 2.5, 5, and 10% EM^®^ for bees, and the control group feed with pure sugar syrup. Each group had five replicates. Cane sugar and drinking water were mixed 1:1 (w:v) and warmed to 40 °C. For the treated groups, 2.5, 5, or 10% of EM^®^ probiotic for bees was added to the sugar syrup (2.5, 5, or 10 g of probiotic/100 g sugar syrup). Bees in cages were fed *ad libitum*. We also put a tube with drinking water into each box. Food consumption was recorded daily and food was freshly prepared every two days. Dead bees were counted daily.

#### 2.2.2. Hemolymph Collection

Sampling of hemolymph was done on day 11 and 15 for immunological parameters and on days 13 and 22 for biochemical parameters. Hemolymph was collected as a pool of 3 to 5 adult bees *per* group, according to the method described in Beebook [49]. Hemolymph was separately collected in vials and Eppendorf tubes for different biochemical analysis (glucose, trehalose, total lipids, total proteins, vitellogenin). All samples were stored at −80 °C until analysis.

#### 2.2.3. Biochemical Parameters

##### Glucose, Trehalose, and Lipid Concentrations

The hemolymph sugar content was determined using a commercially available kit (Glucose (GO) Assay Kit, Sigma-Aldrich, Saint Louis, MO, USA). Briefly, the glucose in a sample is oxidized to gluconic acid and hydrogen peroxide. Hydrogen peroxide reacts with o-dianisidine in the presence of peroxidase to form a colored product. The intensity of a color is proportional to the glucose concentration. The optical density was measured at 540 nm using the spectrophotometer (Shimadzu, Marlborough, MA, USA). The exact amount of glucose was calculated from the standard curve and multiplied by the dilution factor. In the trehalose quantification, the molecule of trehalose was hydrolyzed to two molecules of D-glucose in a reaction catalyzed by enzyme trehalase [49]. The trehalose concentration was calculated as the reading of the proper reaction minus the prior determined glucose concentration in the same sample. The obtained results are multiplied by the molecular weight of trehalose (342.3 g/Mol) and divided by 2× the molecular weight of glucose (180 g/Mol) because the trehalose splits trehalase into two glucose molecules.

Total lipids in the samples were determined by the sulpho-phospho-vanillin method [50]. The hemolymph sample was mixed with 200 µL of sulphuric acid (Sigma-Aldrich, St. Louis, MO, USA) and incubated (10 min, 100 °C). After quick cooling on ice, vanillin was added (2 mL, 13 mM in 66.8% phosphoric acid) (Sigma-Aldrich, St. Louis, MO, USA). Following 30 min of incubation at the room temperature, the optical density was measured at 546 nm using the spectrophotometer (Shimadzu, Marlborough, MA, USA). The concentration of total lipids was calculated from a standard calibration curve obtained from the serial dilution of oleic acid.

#### 2.2.4. Immunological Parameters

##### Concentrations of Total Proteins and Vitellogenin in Hemolymph

The total protein concentration in honey bee’s hemolymph was measured according to the Bradford method (commercial kit from Bio-Rad Hercules, Berkeley, CA, USA). To prevent melanisation of hemolymph, the samples were held on ice during analyses. The optical density was measured at 595 nm using the spectrophotometer (Shimadzu, Marlborough, MA, USA). The exact amount of total protein was calculated from a standard calibration curve prepared from bovine serum albumin (Sigma-Aldrich, St. Louis, MO, USA).

The concentration of vitellogenin (VG) was measured using a Vitellogenin Elisa kit (MyBioSource, San Diego, CA, USA) according to the manufacturer’s instructions. This kit is based on VG antibody-VG antigen interactions (immunosorbency) and an HRP colorimetric detection system. The optical density was measured at 450 nm using the spectrophotometer (Shimadzu, Marlborough, MA, USA). The level of vitellogenin in a sample was calculated from a calibration curve.

#### Hypopharyngeal Gland Size

On the 11th (I) and 15th (II) day of the experiment the honey bees (N = 5 per diet: EM 5%, EM 10%, control) were shortly (~5 min) held in the freezer and then HPGs were dissected and prepared for histological examination and acinar diameter measuring. In group 2.5% EM the HPGs were not analyzed. The HPG tissue was fixated in 10% formaldehyde, dehydrated through a series of ethanol solution (70%, 80%, 90%, 100%; 24 h each), then 100% 2-propanol (24 h), 100% 2-propanol (12 h), 100% 2-propanol (24 h), 2-propanol: Paraffin wax (1:1, 24 h), 3× paraffin wax (24 h each), and embedded in paraffin wax. The wax blocks were cut with microtome (Leica RM2255, Biosystems Nussloch GmbH, Nussloch, Germany) on 7 µm thick cuts and placed on microscopic glass slides. Then, the microscopic slides were stained with Hematoxylin & Eosin [51]. HPGs were measured using the AxioVision 4.6 program (Carl Zeiss, San Diego, CA, USA). Measuring of the HPGs acini diameter was performed on 5 to 24 acini *per* bee and the average diameter per group was calculated.

### 2.3. Statistical Analyses

In order to assess and verify the differences in the spore load data between groups at different sampling dates, the one-way analysis of variance (ANOVA) with Turkey’s Post Hoc test for multiple comparisons and Mann-Whitney U test were performed using the statistical software package GraphPad Prism software version 7.00 for Windows (GraphPad Software, La Jolla, CA, USA). Data were checked for normality using the Shapiro Wilk Test. The results are presented as the mean values and standard deviations. The significance level of α = 0.05 was set to define statistical differences (0.95 confidence interval).

## 3. Results

### 3.1. Strength of Honey Bee Colonies

**Figure 1:**
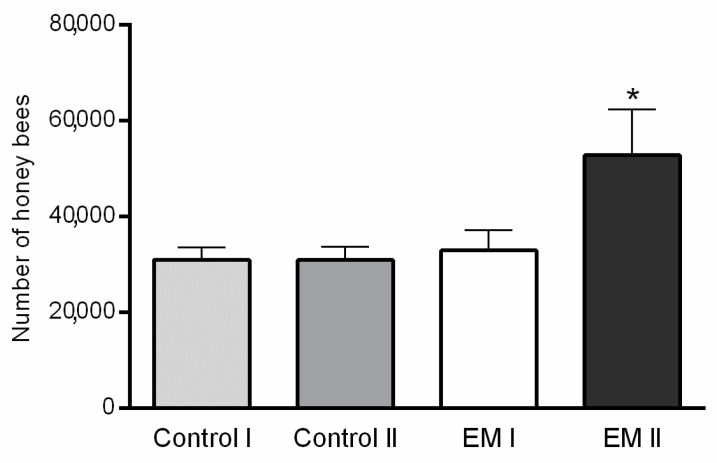
Honey bee colony strength differences between experimental (EM I, EM II) and control (Control I, Control II) groups by estimation dates (I-1st day, II-40th day of the experiment); statistically significant difference: Control II vs. EM II; * *p* < 0.05; mean ± SD.

Variations in the average number of honey bees *per* group during two estimation dates are shown in Figure 1. The statistically significant differences in honey bee colony strength between control and experimental groups were determined on day 40 (*p* < 0.001; F = 17.71). A higher number of bees was estimated in the EM II group compared to control II, while the colony strength of control I and EM I group was similar.

### 3.2. Estimation of Nosema spp. Infection Levels

**Figure 2:**
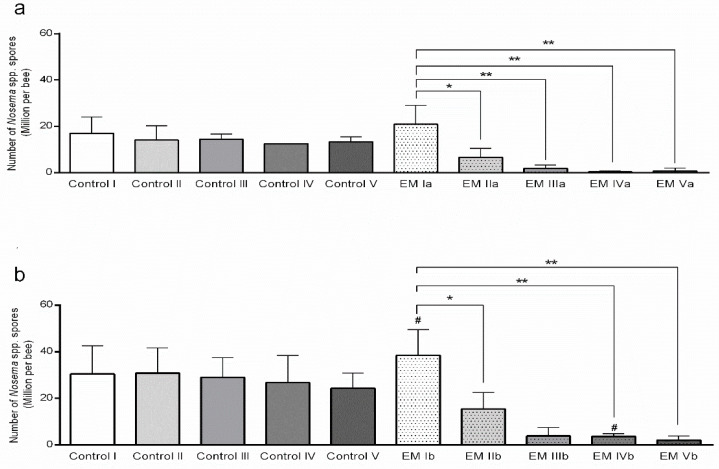
Number of *Nosema* spp. spores *per* honey bee during additional feeding in field conditions; (**a**) food application by spraying, (**b**) food application in feeders. * *p* < 0.01, ** *p* < 0.001 (statistically significant difference between sampling days for experimental group EM); # *p* < 0.01 (Control I vs. EM Ib, Control IV vs. EM IVb), mean ± SD.

A continuous decline in the *Nosema* spp. infection levels in honey bee colonies fed with sugar syrup supplemented with EM^®^ for bees on given sampling dates (days) was confirmed as statistically significant by the ANOVA test (F = 10.12; *p* < 0,0001), as presented in Figure 2. A decline in the number of *Nosema* spp. spores in adult bee samples originated from the honey bee colonies fed with EM^®^ for bees (a, b) on the second sampling date (day 10) (*p* < 0.01), though a statistically lower number of spores in comparison to the initial field experiment day was already observed. A decline in the number of spores was confirmed for each subsequent sampling date, on day 20th (only for a), 30th, and 40th (*p* < 0.001), respectively.

Moreover, in the experimental group a, the reduced numbers of spores compared to the initial spore count at an average of 47.91% on day 10; 92.12% on day 20; 93.49% on day 30, and 91.74% on day 40, were determined. In the experimental group b, the spores reduction counts compared to the initial spore count at an average, were as follows: 67.01% on day 10; 85.3% on day 20; 92.14% on day 30, and 95.66% on day 40 after the initial sampling.

### 3.3. Adult Bees’ Mortality and Consumption of Diets

**Figure 3:**
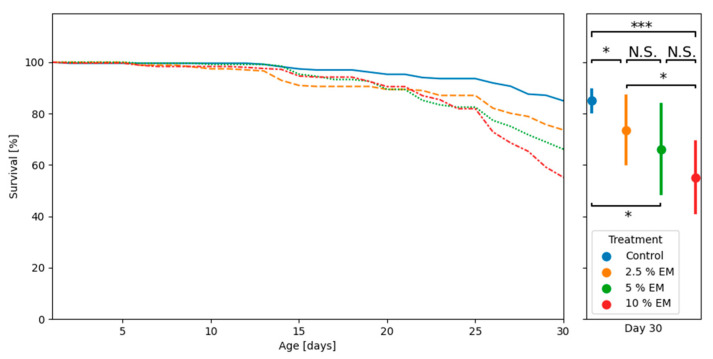
The adult bees’ mortality curves during trials in laboratory-controlled conditions, for control and experimental groups (EM 2.5%, EM 5%, EM 10%); asterisks indicate statistically significant differences: Control vs. EM II vs. 2.5% EM, * *p* < 0.01; Control vs. 10% EM, *** *p* < 0.001; N.S.: No significant differences.

Mortality analyses of adult honey bees during trials in laboratory-controlled conditions revealed significant differences in survival between the two supplemented groups (EM 2.5% and EM 10%), as well as between the mentioned experimental groups and control group (Figure 3).

**Figure 4:**
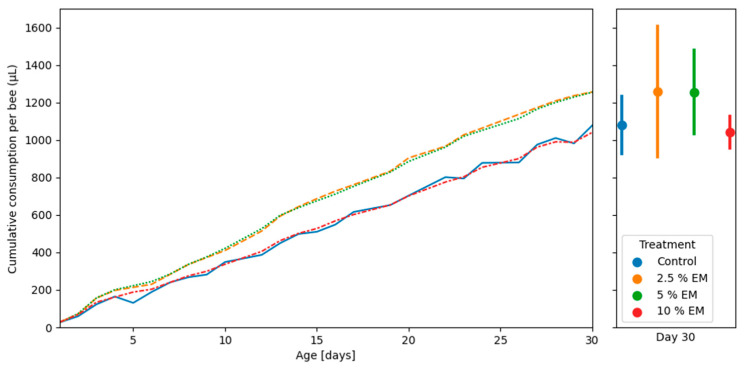
Cumulative amounts of diet consumption during trails in laboratory-controlled conditions, for control and experimental groups (EM 2.5%, EM 5%, and EM 10%). No significant differences.

The honey bees revealed no significant difference in cumulative diet consumption during trials in laboratory-controlled conditions, between the three experimental groups (EM 2.5%, EM 5%, EM 10%), as well as between the mentioned experimental groups and control group (Figure 4). The mean cumulative consumption *per* control group was 1077.58 µL and for experimental groups (EM 2.5%, EM 5%, EM 10%) were 1256.12, 1254.50, and 1040.54 µL, respectively.

### 3.4. Biochemical Parameters

**Figure 5:**
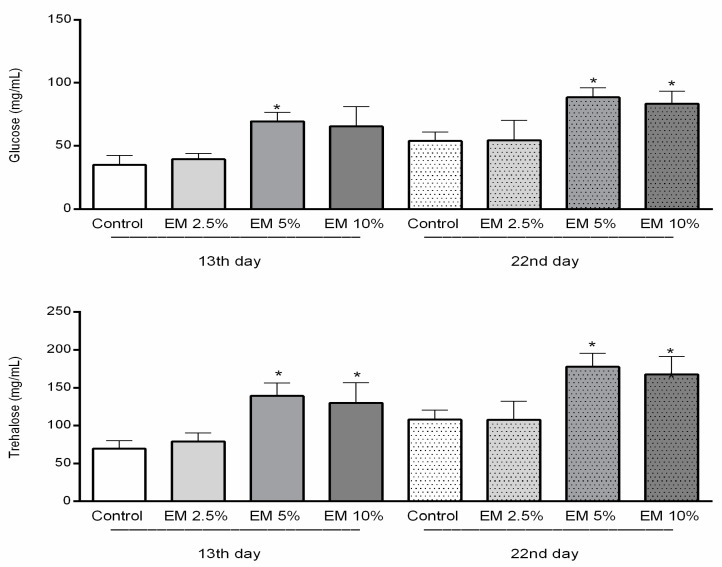
Concentrations of glucose and trehalose in honey bee hemolymph sampled on the 13th and 22nd day of the experiment in laboratory-controlled conditions for control and experimental groups (EM 2.5%, EM 5%, EM 10%). Asterisks indicate statistically significant differences, Control vs. EM 5% and EM 10%; * *p* < 0.001; mean ± SD.

The carbohydrates concentrations were significantly higher in the hemolymph of bees in experimental groups (Figure 5) (F = 14.23; *p* < 0.0001), compared to the control. Moreover, the glucose concentration was significantly higher in experimental groups compared to the control: 13 days old bees from group EM 5% (*p* < 0.01), as well as 22-day-old bees from groups EM 5% and EM 10%. Trehalose concentrations in honey bee hemolymph sampled on the 13th and 22nd day were also higher in EM groups in comparison with their controls (F = 16.55; *p* < 0.0001). In detail, trehalose concentrations in experimental group EM 5% was statistically higher than in the control (*p* < 0.001; *p* < 0.0001), and similar results were obtained for experimental group EM 10% (*p* < 0.01; *p* < 0.0001), in both observed occasions. The carbohydrate concentrations (glucose, trehalose) determined in the control group were relatively stable during the whole studied period.

**Figure 6:**
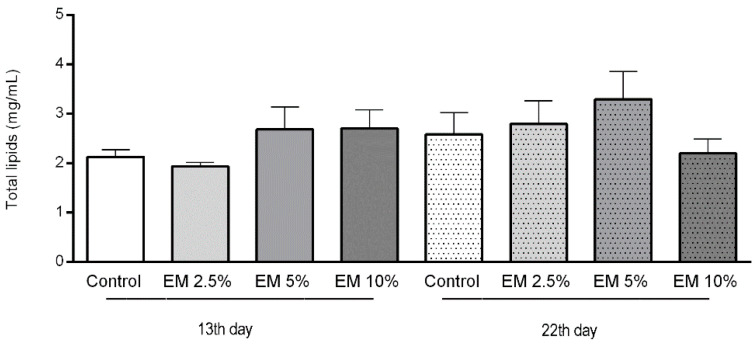
Total lipids concentration in honey bee hemolymph sampled on the 13th and 22nd day of the experiment in laboratory-controlled conditions, for control and experimental groups (EM 2.5%, EM 5%, EM 10%). No significant differences between control and experimental groups were found.

The concentration of total lipids in adult bee’s hemolymph varied during the whole observation period, and there were no significant differences determined among the control and experimental groups (Figure 6). The mean lipid concentrations in hemolymph of 13-day-old bees in the control group were 2.130 mg/µL and experimental groups EM 2.5%, EM 5%, and EM 10% were 1.940, 2.690, and 2.705 mg/µL, respectively. In a second sampling occasion, the lipid concentrations in the control were 2.585 and in experimental groups EM 2.5%, EM 5%, and EM 10% varied as follows: 2.800, 3.300, and 2.200 mg/µL.

### 3.5. Immunological Parameters

The total proteins concentration in hemolymph was significantly higher only in experimental group EM 5% in 15-day-old bees compared with other examined groups.

**Figure 7:**
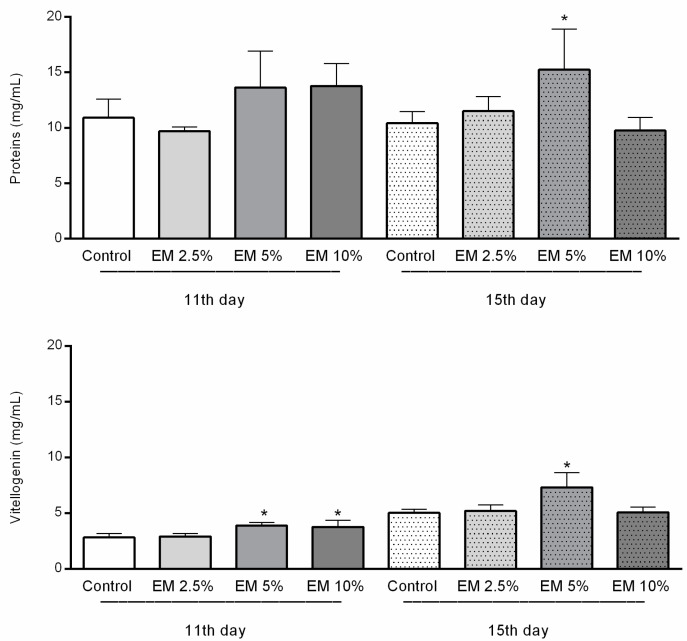
Total proteins and vitellogenin concentrations in honey bee hemolymph sampled on the 11th and 15th day of trail in laboratory-controlled conditions, for control and experimental groups (EM 2.5%, EM 5%, EM 10%). Asterisks indicate statistically significant differences, Control vs. EM 5% and EM 10% on the first and EM 5% on both observed dates, * *p* < 0.01; mean ± SD.

The concentrations of vitellogenin were significantly higher in the hemolymph of adult bees in EM experimental groups, compared with their pertaining controls, but just those fed with addition of EM 5% and EM 10% in the first; and EM 5% in a second sampling date (F = 23.29, *p* < 0.0001, Figure 7). Vitellogenin concentrations in EM 2.5% in the first and in EM 10% in the second sampling occasion were similar to their pertaining controls.

### 3.6. Effect of EM Supplementary Nutrition Regime on Hypopharyngeal Gland Size

**Figure 8:**
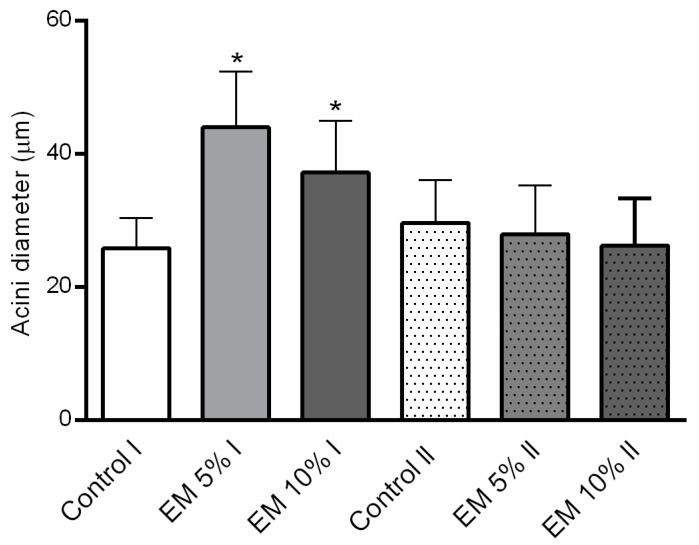
Hypopharyngeal glands acini diameter (µm), dissected from the 11 (I) and 15 (II) days old adult honey bees, during trials in laboratory-controlled conditions, for control (Control) and experimental groups (EM 5%, EM 10%). Asterisks indicate statistically significant differences comparing the mean acini diameters between diets *per* group and represent a * *p* < 0.0001 level of significance; mean ± SD.

The acini diameter of HPGs of 11-day-old bees from EM 5% and EM 10% fed experimental groups was significantly larger than in the bees fed with pure sugar syrup, in the same age (F = 17.41; *p* < 0.0001). No differences were established between the control and experimental groups in 15-day-old bees. Results are shown in Figure 8.

## 4. Discussion

EM^®^ for bees has been successfully used by beekeepers in Croatia and neighboring countries for the last few years. There are no available scientific reports about EM^®^ for bees effects on honey bee colonies life and health. Due to that, we considered that it is crucial to investigate its potential as a therapeutic and immunomodulatory food supplement. To our knowledge, this is the first study aim to investigate the effects of EM^®^ for bees on honey bee colonies health condition and to address some gaps in knowledge of therapeutical, biochemical, and immunological parameters of honey bees exposed to probiotic treatments, in field and laboratory conditions. Except for the beekeepers reports which do not necessarily imply positive effects, there are published controversial records of improper protein supplementation [52,53,54], as well as side effects of the use of some other commercial pro- and prebiotics [55]. Opposite to the last reported results [55], in our experiment conducted in combined field and laboratory conditions, EM^®^ for bees supplement induced a significant decrease in the *N. ceranae* infection level along with increased values of some parameters of the immune system response.

Changes in the honey bee colonies’ strength trends were different between experimental and control groups, but within the expected ranges under the study and environmental conditions. Significantly stronger colonies of the experimental group at the end of the observed period were detected. These results were in accordance with previously published scientific records on multiple feedings with sugar syrup supplemented with herbal extracts [56,57] and using probiotics, mostly of the genera *Lactobacillus* and *Bacillus* isolated from the *A. mellifera* intestines, as well as the human probiotics which stimulates the queen on egg laying [58,59,60,61].

The results of this study demonstrated that EM^®^ for bees administrated via a sugar syrup clearly reduce the development of the microsporidium *N. ceranae* in the honey bee gut. This was observed in the significantly lower spore counts in experimental groups compared to their pertaining control group observed from the initial time point on the 10th, 20th, 30th, and 40th day of the field experiment (Figure 2). It is known that bees invaded with *N. ceranae* and fed with natural beebread show higher microbiota stability and lower mortality rates than those fed only with sugar syrup [62]. Due to this fact, we chose a field experiment where colonies were provided with natural protein food sources, except with additional food. The control honey bee colonies showed lower average reduction of *N. ceranae* spores and were on similar infection levels during the whole experimental period. Slightly better results were obtained for experimental group a, with an application of EM^®^ for bees by the drenching method in comparison to colonies where food was offered in feeders (b), what can be explained with more efficient food supplement distribution on the account of adult bees’ social behavior [63]. Other scientific reports regarding the ability of different bacterial strains to suppress the infection level of *N. ceranae*, after oral administration, also showed positive results, e.g., effects of *Parasaccharibacter apium* [64], *Bifidobacterium,* and *Lactobacillus* strains [38] in laboratory conditions; and *Bacillus subtilis* and *Lactobacillus johansonii* under realistic field conditions [59,60]. Except for good results in the nosemosis growth inhibition, the beneficial effects linked with the queen’s production, reducing of *V. destructor* mites number [65], increased honey production [59,65,66], and increase of larvae and adult bees’ viability [34,67] were reported. Given that analyzing the percentage of forager bees infected by *Nosema* spp. spores was previously demonstrated to be a more accurate method of estimating the level of infection at the colony level [68], we applied this method to examine the therapeutic capacity of EM^®^ for bees as a dietary supplement. According to the results presented in this study, and the excellent results in other studies on biological and therapeutic effects for different disorders in animals, plants, and the environment [69,70,71,72,73], it should be acknowledged that EM^®^ holds a substantial potential for nosemosis treatment.

Mortality rate analyses of adult honey bees during trials in laboratory-controlled conditions revealed a significant difference between control and experimental groups, especially for 10% EM. The results related with survival rates of bees in laboratory conditions reported by Maistrello et al. [74] and Arredondo et al. [34] were more advantageous, in comparison with our results. Observations at apiary conditions indicate the absence of negative impacts of EM^®^ for bees on brood and adult bee’s health and survival.

We found that adult bees originating from the experimental groups during trials in laboratory-controlled conditions consumed similar amounts of the food compared to the control groups. On the contrary, previous studies reported that bees ate higher amounts of the supplemented food [75].

In our study, the levels of carbohydrates (glucose, trehalose) were statistically higher in the experimental groups of honey bee colonies. Usually, the concentrations of carbohydrates are stable during the year, but they rapidly increase when hemolymph sampling is conducted the day after autumn’s administration of sugar syrup in hives [76]. Moreover, some authors suggest that due to different sugar degradation pathways the beneficial microbes could more successfully colonize a sugar rich digestive system in bees [77]. In accordance, we found the lipid content to be very stable during both hemolymph sampling dates with no significant differences between control and experimental groups, not surprisingly in respect to the carbohydrate diet of caged honey bees in laboratory conditions. Those results were contrary to results of Chakrabarti et al. [75].

Nevertheless, despite the diet type, concentrations of proteins were increased in the EM 5% experimental group, 15 days after the experiment in control laboratory conditions started. Those results are in accordance with previous reports on the impact of dietary sterols on various fitness traits in insects [75]. Although the vitellogenin key role is reproduction, except in the queen, it can be detected at high concentrations in sterile workers hemolymph [78]. As vitellogenin hemolymph titers are linked with honey bee immunity [79] and are able to bind to multiple pathogens [80], we chose to investigate their concentration in the hemolymph of adult bees that originate from experimental and control groups. Moreover, considering the fact that beneficial microorganisms could stimulate the host’s immune response and improve the resistance to diseases [81] is especially important because infection with *N. ceranae* can depress the honey bee immune system [82]. In our study, vitellogenin concentrations in hemolymph sampled on the 11th and 15th day of trials in laboratory-controlled conditions showed a significant increase in group EM 5% and EM 10% on the first and EM 5% on both observed occasions, compared with their controls. Despite the finding that the increased HPG size does not always represent a good physiological status [13] or even appears as a result of negative effects of nutritional stress and dysbiosis [19], we noticed an increase in HPG size and vitellogenin concentration in younger bees that were fed with the EM probiotic. The results can be explained by the fact that the physiological status of bees reacted positively with the presence of beneficial microbes in the food, taking into consideration that HPGs host bacterial communities respond differently depending on the food quality [19]. In addition, there are changes in bacterial loads and community composition in different seasons and tasks of bees (nurses, foragers, winter bees) due to the different food [83] that is available in nature or stored in the hive.

In practice, the beekeepers treat honey bee colonies against varroosis using veterinary medicines. In the case of infection with another pathogen including *Nosema* spp., the careful use of commercial probiotics with beneficial microorganisms, considering their metabolites production, may represent a natural tool for protecting or reducing the load with pathogens in honey bees. In our opinion, this study shows the possible role of beneficial bacteria supplementation in supporting honey bee health as a preventive measure and new tool for the management of the bee microbiome. Further studies are necessary to investigate the efficacy of the EM probiotic on honey bee colonies in different seasons and evaluate possible effects on honey production.

## 5. Conclusions

Due to the fact that numerous negative factors contribute to a decline in insect pollinators, it is fundamental to research all the nutritional requirements crucial to improve and sustain the honey bee colonies health. EM^®^ for bees is a promising alternative food additive for nosemosis combating in honey bee colonies causing consecutive reductions in spore counts after proper *in hive* administration. Overall, this is the first study reporting the revealed novel insights about the EM^®^ for bees uptake benefits and its role in honey bee nutritional physiology.
